# Cod-Liver Oil in Scrofulous Affections and in Consumption

**Published:** 1851-09

**Authors:** 


*Cod-Liver Oil in Scrofulous Affections and in Consumption.*—Dr. Hays stated to the Philadelphia College of Physicians (February 6, 1851,) that he had employed the cod-liver oil extensively, in the Wills' Hospital and in private practice, during the last three years, in scrofulous ophthalmia, in cases of granular lids, in scrofulous enlargement of the external glands, in cases of hip disease, and in the various forms of external scrofula, with the best effects. In scrofulous ophthalmia he had found it of all remedies the most efficacious. Under its use the constitution becomes invigorated; the glandular swellings are dissipated; and the cutaneous affection so commonly met with about the face and ears disappears. He has also employed it in several cases of granular lids with the most favorable results. In this affection, patients are very liable to relapse, from slight causes; this tendency he has found to be removed by the use of the cod-liver oil, alone, or in conjunction with the syrup of the proto-iodide of iron. In the case of a lad now under treatment affected with scrofulous enlargement of the cervical glands, chronic conjunctivitis, and granular lids, with deposit of lymph in the cornea, and intense photophobia, by the use of the cod-liver oil and proto-iodide of iron, with the occasional application to the eye of the liquor plumbi, all the symptoms of disease are rapidly disappearing. The patient can bear the light without inconvenience, can read small print, and has all the general appearance of restored health. He has escaped a relapse now for four months. The photophobia has disappeared entirely. In another case of excessive photophobia, with granular lids and penetrating ulcer of the cornea, the cod-liver oil has been used (at the suggestion of the *interne* of the Wills' Hospital, Dr. McIntyre), with the most decided advantage.

Dr. Hays has now employed the cod-liver oil in from two hundred to two hundred and fifty cases of scrofulous ophthalmia and granular lids, and in most of these cases the benefit resulting from its use has been very striking.

Dr. Condie remarked that he had employed the cod-liver oil pretty extensively. In all the forms of external scrofula, including scrofulous ophthalmia, he had certainly seen much good result from its use; the indications of strumous disease have very generally ulimately disappeared under its use, while the appetite of the patients has improved, and they have speedily exhibited increase of strength and bulk. It is especially in the scrofulous affections of young subjects that the cod-liver oil had appeared to him to produce the greatest amount of benefit. He had not, however, seen any very striking amount of good result from its administration in cases in which tuberculous deposits had actually taken place in any of the tissues. In tubercular phthisis especially he had rarely, if ever, observed any positive benefit from its use. He had given it in many cases, and in large quantities, and though it had been taken perseveringly by the patients for a long period of time, he could not say that in a single instance the disease of the lungs had been arrested by it. The onward march of these cases towards a fatal termination appeared to him to be as rapid as those in which the oil had not been given.

The wife of a gentleman connected with the public press, now under the care of Dr. C., had used it daily for the last three years, and she believes with very decided advantage. But though in her case the affection in the lungs is of a very chronic character, still the frequent attacks of hemoptysis to which she is subject, and the physical signs revealed by auscultation, indicate, but too clearly, that her disease is making a slow but constant progress.

Dr. Wood said that he should not be doing justice to his own feelings were he not to seize this opportunity of communicating to the College the information on the subject under consideration he had derived from his recent experience. He felt himself the more strongly urged to make this communication from the fact, that in the last edition of his work on the Practice of Medicine, he had stated that the only effect he had observed from the use of cod-liver oil was the production of nausea. Now the reason of this he had ascertained was that he had not persevered sufficiently long with the use of the remedy. Subsequently he had employed it largely and perseveringly, in hospitals as well as in private practice, and most assuredly he had never met with any one remedy or combination of remedies which proved so efficacious as this in pulmonary phthisis. It is too soon, however, to say that in any case it will permanently cure the disease. He has certainly seen cases presenting, apparently, all the phenomena—the general symptoms as well as the physical signs—characteristic of phthisis, get well under its use. The patients have, at least, lost their cough and fever, their respiration has become natural, and they have acquired strength and flesh. In one case which occurred in the hospital, auscultation indicated the existence of a cavity at the summit of one lung, and deficient respiratory murmur in the other. The patient was pale, extremely feeble and emaciated, with hurried respiration, cough, and copious expectoration, hectic fever, and night sweats. Although in this case Dr. W. expected little, even temporary benefit to result from any plan of treatment, still he thought it his duty to place him under the use of the cod-liver oil. At the end of six or eight weeks, he was surprised to see the patient able to sit up, and considerably improved in all respects. From that time the unfavorable symptoms gradually lessened, the cough and expectoration abated, the respiration more free and easy, the hectic fever and night sweats disappeared, the pulse increased in strength and diminished in frequency; and, at the termination of four months from the commencement of the treatment, the patient had become fat, ruddy, strong, and, to all appearance, entirely well. Upon an examination of the chest, a deficiency of respiration was detected in the vicinity of that portion of the lung in which a cavity had existed—which is precisely what we should have anticipated. This is the most striking case which he has met with of the cure of pulmonary disease under the use of cod-liver oil.

The natural cure of tubercle is by the tuberculous matter becoming softened, and then discharged by expectoration. Now, if by any means we can so far modify the nutritive process as to prevent a further deposition of tuberculous matter, we may have a reasonable hope of a cure taking place in many cases of tubercular phthisis. We have the record of instances where this has actually taken place; the cicatrices of the discharged vomicæ being apparent, after death from other causes, many years subsequent to the disappearance of the symptoms indicative of phthisis.

In what had appeared to be the incipient stage of consumption, Dr. Wood had seen several very striking instances of the efficacy of cod liver oil. In one case, that occurred in a child, he was convinced that there existed a cavity in one of the lungs. So far as the physical signs derived from auscultation were to be depended on, its presence appeared to be established.—The patient was emaciated, had cough, impeded respiration, and hectic fever. Dr. Wood had some apprehension that it was a case of pulmonary tuberculosis. The child was put upon the use of cod-liver oil, with a proper diet and regimen, and at the end of six weeks the symptoms of disease had so far disappeared that the patient was considered convalescent. He ultimately recovered.

A middle-aged gentleman, who had suffered from repeated but slight attacks of hemorrhage from the lungs, became, subsequent to one of these attacks, emaciated and enfeebled in strength, with a frequent pulse and obstinate cough. Auscultation revealed, no positive indications of pulmonary disease; but the general symptoms were such as to occasion much solicitude. Under the use of cod-liver oil, and at the termination of six weeks, he began to improve, and finally recovered.

In another case, that of a young man about twenty years old, the patient, who was convalescent from an attack of typhoid fever, became affected with cough, very frequent pulse, expectoration, fever, night sweats, and emaciation. Auscultation of the lungs evinced the probable existence of tuberculous deposition at the summit. Dr. W. considered this patient in imminent danger, unless he should be able to bring to bear upon his case influences more beneficial than those he had formerly had at his disposal. The patient was placed upon the use of cod-liver oil, with an appropriate diet and regimen; and was sent on a voyage to Europe. At the end of about six weeks he began to improve; and, on his return, at the end of three or four months, had lost all the symptoms of the disease, except frequent pulse, which gradually diminished under the continued use of the oil; and when Dr. Wood last saw him he was apparently in perfect health.

Here are three cases, in all of which he considered the symytoms under which the patients laboured to indicate disease of the lungs, of a very critical, and in one, almost hopeless, character. Under the use of cod-liver oil they all entirely recovered—so far at least as a disappearance of the morbid symptoms and a return of strength and vigour can indicate an entire recovery. He was not prepared to say that they were all instances of pulmonary tuberculosis—though there were strong reasons for believing that in most of them such was the case. They indicate, however, the very excellent effects to be derived from the oil in cases of a very troublesome and threatening character. It is important that the use of the remedy should be persevered in for a length of time. There is much, too, sold for cod-liver oil which contains little or none of the genuine article. If it has a strong taste and smell of boot-leather, it may be considered genuine.

Dr. Hays remarked that the communication just made by Dr. Wood was particularly valuable, and furnishes strong testimony in favor of cod-liver oil as a remedy in phthisis. His own experience of the effects of the article in that disease was but slight and by no means so favorable as that of Dr. W. In one case, however, the oil appeared to him to prove decidedly beneficial. It was that of a boy five years old, laboring under a violent cough, which resisted all the usual remedies. During a paroxysm of crying the patient was attacked with hemorrhage from the lungs, which recurred six times within ten days. In one of these attacks Dr. H. was assured that the child had discharged half a pint of blood.

The cough now became more constant and troublesome; the pulse rapid and feeble; there was expectoration of purulent matter, great restlessness, hectic fever. and excessive prostration. The child was put on the use of cod-liver oil, with a pectoral mixture to relieve the cough. The patient gradually acquired strength—the cough and expectoration diminished—the appetite returned, and health was finally restored. Since his recovery he has gone through an attack of pertussis without a recurrence of his pulmonary disease, and he now appears to be perfectly well.

Dr. H. has administered the cod-liver oil in cases of phthisis occurring in adults without any benefit—so far as the arrest of the disease was concerned—though he had no doubt the remedy even in these cases was often productive of good by its influence in improving nutrition. —*Quarterly Summary of the Tran. of the Philad. Col. of Phys.*, vol. i. No. 2, N. S.

